# Using Machine Learning Methods to Predict Cognitive Age from Psychophysiological Tests

**DOI:** 10.3390/healthcare13243193

**Published:** 2025-12-05

**Authors:** Daria D. Tyurina, Sergey V. Stasenko, Konstantin V. Lushnikov, Maria V. Vedunova

**Affiliations:** 1 Institute of Biology and Biomedicine, Lobachevsky State University of Nizhniy Novgorod, Gagarin Avenue 23, 603022 Nizhny Novgorod, Russia; 2 Moscow Center for Advanced Studies, 20 Kulakova Str., 123592 Moscow, Russia

**Keywords:** machine learning algorithms, cognitive test, human age, data analysis

## Abstract

**Background/Objectives:** This paper presents the results of predicting chronological age from psychophysiological tests using machine learning regressors. **Methods:** Subjects completed a series of psychological tests measuring various cognitive functions, including reaction time and cognitive conflict, short-term memory, verbal functions, and color and spatial perception. The sample included 99 subjects, 68 percent of whom were men and 32 percent were women. Based on the test results, 43 features were generated. To determine the optimal feature selection method, several approaches were tested alongside the regression models using MAE, $R^{2}$, and $CV\text{\_}R^{2}$ metrics. SHAP and Permutation Importance (via Random Forest) delivered the best performance with 10 features. Features selected through Permutation Importance were used in subsequent analyses. To predict participants’ age from psychophysiological test results, we evaluated several regression models, including Random Forest, Extra Trees, Gradient Boosting, SVR, Linear Regression, LassoCV, RidgeCV, ElasticNetCV, AdaBoost, and Bagging. Model performance was compared using the determination coefficient ($R^{2}$) and mean absolute error (MAE). Cross-validated performance ($CV\text{\_}R^{2}$) was estimated via 5-fold cross-validation. To assess metric stability and uncertainty, bootstrapping (1000 resamples) was applied to the test set, yielding distributions of MAE and RMSE from which mean values and 95% confidence intervals were derived. **Results:** The study identified RidgeCV with winsorization and standardization as the best model for predicting cognitive age, achieving a mean absolute error of 5.7 years and an $R^{2}$ of 0.60. Feature importance was evaluated using SHAP values and permutation importance. SHAP analysis showed that stroop_time_color and stroop_var_attempt_time were the strongest predictors, followed by several task-timing features with moderate contributions. Permutation importance confirmed this ranking, with these two features causing the largest performance drop when permuted. Partial dependence plots further indicated clear positive relationships between these key features and predicted age. Correlation analysis stratified by sex revealed that most features were significantly associated with age, with stronger effects generally observed in men. **Conclusions:** Feature selection revealed Stroop timing measures and task-related metrics from math and campimetry tests as the strongest predictors, reflecting core cognitive processes linked to aging. The results underscore the value of careful outlier handling, feature selection, and interpretable regularized models for analyzing psychophysiological data. Future work should include longitudinal studies and integration with biological markers to further improve clinical relevance.

## 1. Introduction

Cognitive decline associated with aging is becoming a serious public health concern. Research in the field of healthy longevity is becoming increasingly prominent. By 2050, at least 152.8 million people worldwide are projected to suffer from dementia [1]. Interest in diagnosing human cognitive and physiological functions during the natural aging process is growing. Treatments for cognitive impairment and dementia exist, but progress in this area has been limited [2]. Therefore, additional methods are needed for both the early diagnosis of cognitive abilities and interventions that will help reduce the personal, social, and economic costs associated with the increasing number of dementia diagnoses [3].

Many scientists agree that chronological, biological, and cognitive age often do not correspond to one another [4]. Biological age refers to the actual state of the body, rather than the number of years lived (chronological age). Cognitive age refers to the degree of preservation or decline in processes such as memory, attention, information processing speed, language abilities, and visuospatial skills. Modern science aims to reduce—or at least slow—the rate of aging in both biological and cognitive terms [5,6]. Achieving these objectives requires accurate diagnostics. Determining biological and cognitive age requires an average aging curve, identified through biomarkers in the case of biological age and psychological or psychophysiological indicators for cognitive age. This curve can then be used to determine the relative difference between an individual’s indicators and the average indicators for a given chronological age. Although this approach is promising, challenges remain. A recent analysis of more than 30 articles published between 1996 and 2022 on determining biological age showed that no single biomarker has a reliably strong correlation with biological age. In this regard, it is crucial to identify biomarkers that most accurately reflect the relationship with chronological age [7]. The concept of brain age offers an intermediate indicator between biological and cognitive age. It uses data on functional brain activity (including cognitive functions) to approximate biological age. Numerous mathematical models have been developed in this context, addressing both healthy and pathological aging [8,9]. However, the quality of such modeling depends heavily on the selection of parameters that form the basis of the predictive model. Thus, in the case of cognitive age, the problem remains equally pressing and centers on identifying reliable markers of cognitive function.

Aging is a complex natural process that results in a nonlinear decline in cognitive function [10]. Some older adults retain a sufficient level of cognitive ability, while others experience varying degrees of decline, which can lead to pathological conditions [11,12]. Some researchers believe that cognitive ability peaks around age 30 and then gradually declines; others point to a sharp decline between the ages of 50 and 60 [13,14,15,16,17,18]. Still, others argue that the decline occurs gradually, without a clearly defined age threshold [19,20,21,22].

Considering that different types of information are processed by different cognitive systems and are subject to aging to varying degrees [23,24,25,26,27], constructing an accurate model requires a variety of tests and a large amount of information. Moreover, the results of psychological and psychophysiological tests exhibit significant variability [28,29].

Each psychological and psychophysiological test task activates a large but limited number of brain regions in various combinations. Therefore, it is impossible to assert definitively that each test reveals activity in a specific brain region. Similar examples have been documented for numerous other tests [30,31,32,33].

This study aims to identify the smallest possible set of tests and corresponding indicators that best reflect the state of cognitive brain function. This will reduce the time required to assess cognitive function based on chronological age while maintaining acceptable accuracy. We selected six psychophysiological tests that, in our view, are most likely to correlate with age-related changes. For each test, we sought to identify as many features as possible (43 in total) to determine which would best reflect the relationship with chronological age. Of the features considered, some of them may be linearly related to age, which will allow us to identify the most related set of features and construct an interpretable model that can predict age.

## 2. Methods

### 2.1. Data Collection

During data collection, subjects were asked to complete a questionnaire and psychophysiological tests using the data collection system available at https://dev.cogni-test.ru/ (accessed on 1 October 2025). The psychophysiological test data collection system is a client-side web application built using JavaScript technology, which minimizes delays during testing [34]. It allows individual users or groups of subjects to complete the tests. The system does not support random test ordering—users complete the tests in a strictly predetermined sequence. It does not collect or store any personal data. Participants complete the tests anonymously, using only a numeric identifier (user_id), which is not linked to real names, email addresses, or any other personal information. The data (answers and reaction times) are stored in a SQLite database and analyzed separately from the server as part of the scientific research process.

All tasks (except instructions) are timed, with durations ranging from 3 to 15 s. Timing is tracked on the client side using JavaScript. The transition to the next test occurs automatically, with no option to return to the previous one. The system logic enforces a session limit of 15 min for completing all tests. If a user becomes “stuck” (for example, by stepping away for an extended period), access to the current tests is blocked once the 15-min limit is reached. Any subsequent attempt to submit a response or proceed further results in redirection to the authorization page.

Thus, the system provides a rigid, linear structure: registration → authorization → test completion. There is no option to exit or review progress—the system is designed for a single, uninterrupted completion of the entire series within the allotted time. Data from the database can be downloaded in CSV format.

When registering on the portal https://dev.cogni-test.ru/ (accessed on 1 October 2025), all subjects were informed about the study and provided informed consent. An example of the consent form is available both during registration on the portal and in the Supplementary Materials of this article. This study was conducted in accordance with the Declaration of Helsinki and was approved by the Ethics Committee of Lobachevsky University (Protocol Number 3, dated 8 April 2021).

### 2.2. Description of Tests

Table 1 provides a brief description of the tests and the variables for each subject.

### 2.3. Data Preprocessing

The sample included 99 subjects, 68 percent of whom were men and 32 percent were women. The dataset contained 44 variables, one of which represented age, and one was a categorical variable indicating gender (a detailed table of variables with statistics is provided in Table A1 of the Appendix A). Most variables had numerical values, except for gender. For further analysis, gender was one-hot encoded using the pandas get_dummies function. It was encoded as a binary variable, sex_male, where 1 represents male and 0 represents female. Distributions of the remaining variables across tests are presented in the Appendix A (from Figure A2, Figure A3, Figure A4, Figure A5), Figure A6 and Figure A7.

For each feature, the percentage of outliers was calculated using the following equation:

(1) $$\mathrm{Percentage}\;\mathrm{of}\;\mathrm{outliers}=\frac{\mathrm{Number}\;\mathrm{of}\;\mathrm{outliers}}{\mathrm{Total}\;\mathrm{number}\;\mathrm{of}\;\mathrm{feature}\;\mathrm{values}}\times 100\%$$

**Figure 1:**
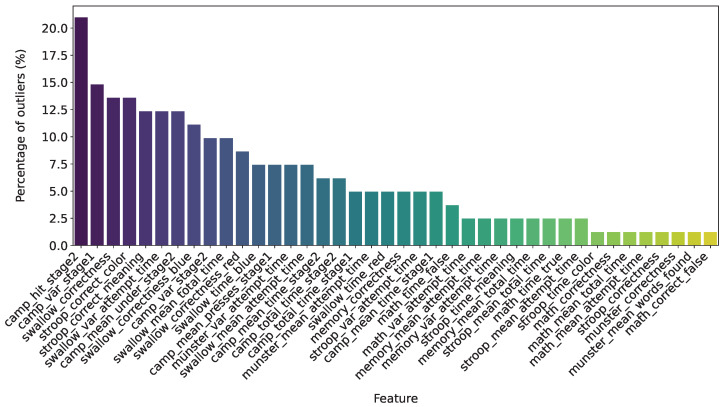
Visualization of the percentage of outliers by feature.

The procedure for identifying outliers in the data is based on the interquartile range (IQR). For each numerical feature, the 25th percentile (Q1) and the 75th percentile (Q3) are first calculated using the quantile method: Q1 = column.quantile (0.25), Q3 = column.quantile (0.75). The interquartile range is then computed as the difference between Q3 and Q1:IQR=Q3−Q1. Next, the lower and upper boundaries for detecting outliers are determined: lower boundary = Q1−1.5×IQR, upper boundary =Q3+1.5×IQR. Values falling outside these boundaries are considered outliers (see Figure 1).

Two strategies were used to handle outliers: the winsorization procedure [35,36] available in the scipy.stats library (the winsorize function), and the RobustScaler function from the sklearn library.

**Winsorization method:** Unlike the classic outlier detection procedure using the interquartile range (IQR), where boundaries are defined as Q1−1.5×IQR and Q3+1.5×IQR, winsorization applies fixed percentile limits: the 5th and 95th percentiles [35,36]. For each numerical feature, values below the 5th percentile are replaced with the 5th percentile, and values above the 95th percentile are replaced with the 95th percentile. This approach ensures that statistics and models are robust to extreme outliers without removing data, thereby preserving the sample size [35,36].

(2) $$x_{\mathrm{winsor}}=\left\{\begin{array}{cc} Q_{0.05}, & \mathrm{if}\;x<Q_{0.05}\\ x, & \mathrm{if}\;Q_{0.05}\leq x\leq Q_{0.95}\\ Q_{0.95}, & \mathrm{if}\;x>Q_{0.95} \end{array}\right.$$

where $Q_{0.05}$ and $Q_{0.95}$ are the 5th and 95th percentiles of the original data, respectively.

**RobustScaler method:** The RobustScaler scales data based on the median and interquartile range (IQR), making it robust to outliers and extreme values. This method can be used as an alternative to the winsorization procedure.

The distribution of the variables shows that their values can differ by orders of magnitude, making a standardization procedure necessary. It is worth noting that although tree-based algorithms are invariant to the scale and range of features, data standardization is required when they are used in conjunction with scale-sensitive algorithms, such as gradient descent or SVM [37,38].

Data standardization was performed using two approaches from sklearn library: StandardScaler and RobustScaler.

**StandardScaler:** Uses the mean and standard deviation to transform the data (Z-score):

(3) $$z=\frac{x-\mu }{\sigma }$$

This approach is sensitive to outliers, as extreme values can distort the calculations of the mean (μ) and variance (σ).

**RobustScaler:** Uses the median and interquartile range (IQR), ensuring robustness to outliers:

(4) $$z=\frac{x-\mathrm{median}}{IQR}$$

Standardization and winsorization were performed only on the training set and subsequently applied to the test set to prevent data leakage.

### 2.4. Machine Learning Algorithms

To predict subjects’ age based on the results of psychophysiological tests, the following regression algorithms were used: Random Forest Regressor, Extra Trees Regressor, Gradient Boosting Regressor, Support Vector Regression (SVR), Linear Regression, Lasso Cross-Validated (LassoCV), Ridge Cross-Validated (RidgeCV), Elastic Net Cross-Validated (ElasticNetCV), Adaptive Boosting Regressor (AdaBoost), and Bagging Regressor (Bagging). The best algorithm was selected based on the values of the determination coefficient ($R^{2}$) and mean absolute error (MAE) metrics.

To split the data into training and test sets, we used the train_test_split function from the scikit-learn library. This function randomly splits the data so that 20% of all records are in the test set, and the remaining 80% are in the training set. The random_state parameter was set to 42 to fix the random number generator for reproducibility of the experiments.

The cross-validated $R^{2}$ (CV_R2) metric was computed using 5-fold cross-validation, where the final score represents the mean of the $R^{2}$ values obtained from all validation folds.

The bootstrap method was implemented as follows: subsamples of the same size are repeatedly generated from the test sample (typically 1000 iterations) using random sampling with replacement. For each subsample, model predictions are obtained, and performance metrics (e.g., MAE, RMSE) are calculated. The distribution of metric values across all subsamples is then compiled, from which the mean and confidence interval (typically 95%) are derived. This approach allows assessment of the dispersion and stability of the metrics without assuming a specific distribution, while accounting for data variability. Ultimately, bootstrapping provides a robust and reliable performance evaluation with uncertainty.

### 2.5. Numerical Simulation Framework

The following Python (version 3.11) libraries were used for data analysis and regression modeling: pandas 2.0.3, numpy 1.22.3, matplotlib 3.7.5, seaborn 0.12.2, scipy 1.10.1, scikit-learn 1.0.2, statsmodels 0.13.5, and shap 0.40.0.

## 3. Results

In our analysis, we first evaluated the performance of two outlier-handling approaches (winsorization and RobustScaler) in conjunction with multicollinearity-resistant regression models (RidgeCV and ElasticNetCV), before proceeding to examine multicollinearity. The winsorization method was applied together with standardization using the StandardScaler from the sklearn library. The results for the two data-processing and standardization approaches show differences in the performance of the RidgeCV and ElasticNetCV models (Table 2).

The model results for the winsor_standard method show that RidgeCV achieves the best performance, with a mean absolute error (MAE) of 7.5460 and ($R^{2}$ = 0.5575), while the cross-validation value ($CV\text{\_}R^{2}$ = −0.5868), indicating some overfitting or model instability when evaluated on the validation data. ElasticNetCV in this case shows an MAE of 8.5215, ($R^{2}$ = 0.3977), and ($CV\text{\_}R^{2}$ = −0.0536), which is worse than RidgeCV but more stable.

For robust scaling, RidgeCV yields slightly worse metrics: MAE = 7.6580, ($R^{2}$ = 0.5169), but ($CV\text{\_}R^{2}$) deteriorates sharply to −242.4276, indicating extremely poor model performance during cross-validation. ElasticNetCV performs even worse, with MAE = 11.3410, negative ($R^{2}$ = −0.1227), and ($CV\text{\_}R^{2}$ = −0.5374), demonstrating poor model performance without additional preprocessing.

Thus, preprocessing via winsorization and standardization (winsor_standard) with RidgeCV provides more stable and better results compared to purely robust scaling. ElasticNetCV exhibits less stability, especially on data processed with only robust scaling.

### 3.1. Removing Multicollinearity from Data

**Figure A1:**
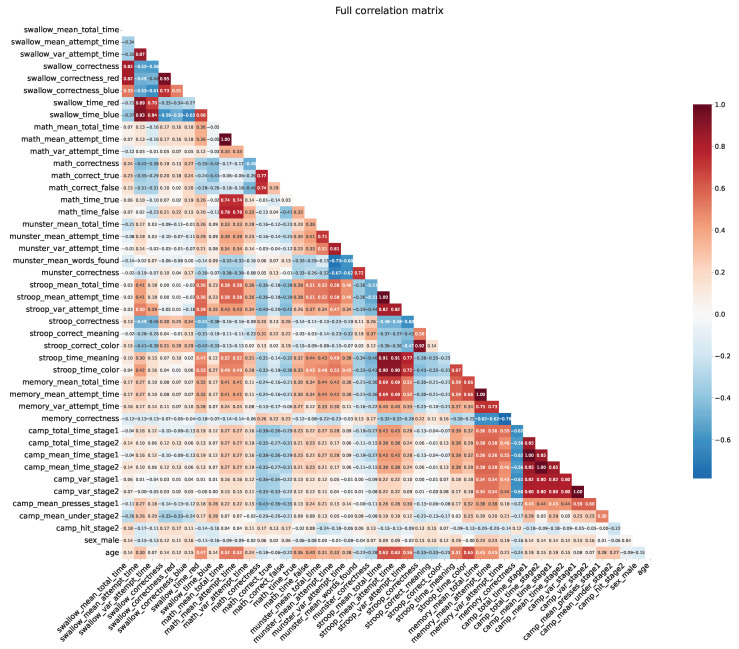
Triangular correlation matrix of the data variables.

**Figure A2:**
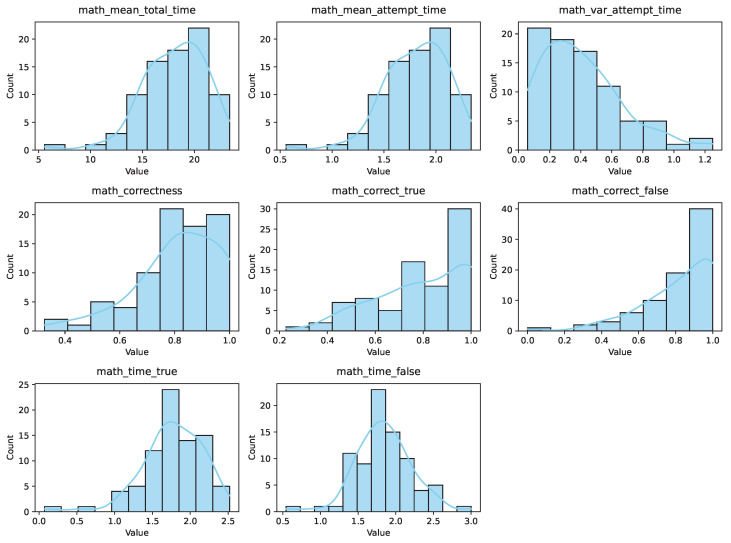
Distribution of variable values for the reaction speed test.

**Figure A3:**
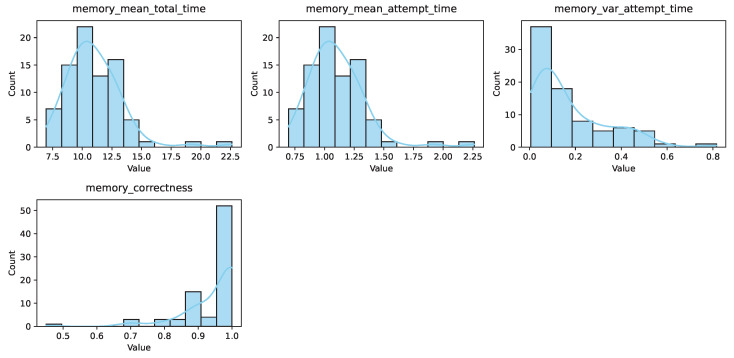
Distribution of variable values for the verbal memory test and working memory capacity.

**Figure A4:**
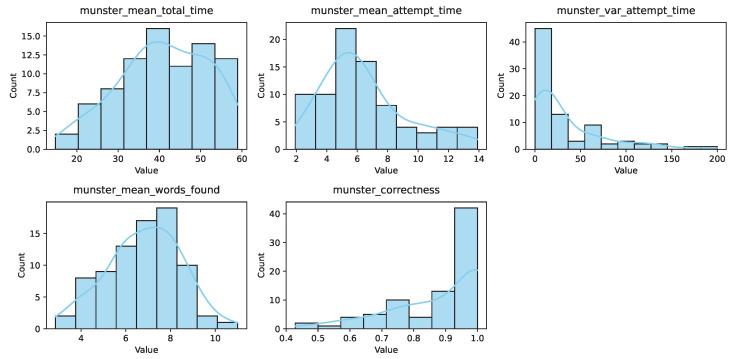
Distribution of variable values for the verbal function test.

**Figure A5:**
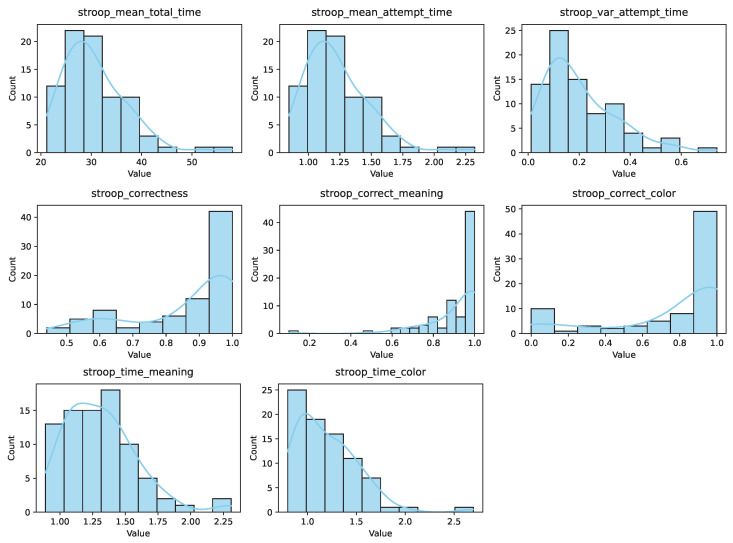
Distribution of variable values for the decision-making ability test.

**Figure A6:**
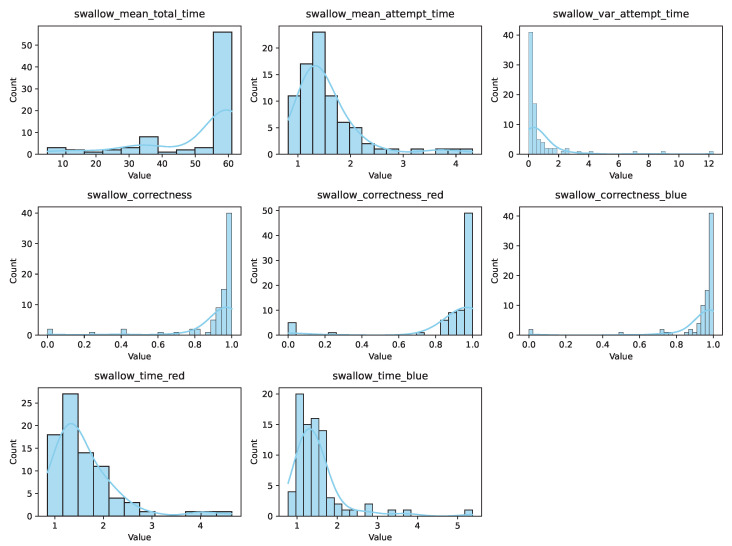
Distribution of variable values for the spatial perception test.

**Figure A7:**
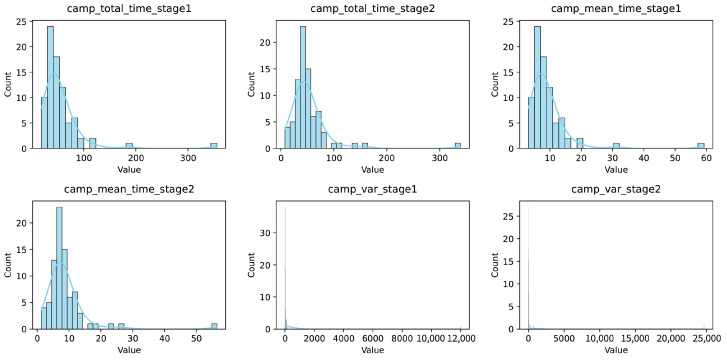

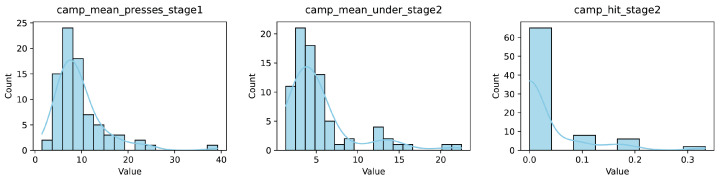
Distribution of variable values for the campimetry test.

Multicollinearity is known to distort parameter estimates by increasing the instability and dispersion of coefficients, particularly in interpretable regression models, making it difficult to isolate the influence of individual features [39]. To examine the data for multicollinearity, a correlation graph was generated using Pearson’s correlation coefficient, computed with the built-in corr() method from the pandas library with default parameters. Since our dataset contains 44 variables, including the target (age), a triangular correlation matrix was used to better visualize the collinearity of the variables (Figure A1).

Figure A1 presents a triangular correlation matrix heatmap summarizing the relationships among multiple cognitive and sensorimotor variables derived from various behavioral tasks, including the Reaction Speed Test, Verbal Function Test, Verbal Memory and Working Memory Capacity Test, Decision-Making Test, Color Campimetry Test, and Spatial Perception Test, as well as the variable Age. Only the lower half of the matrix is visualized to avoid redundancy, as a correlation matrix is symmetric. The heatmap uses a diverging color scale ranging from negative to positive values, typically with blue representing negative correlations and red or orange representing positive correlations. The dataset includes approximately 43 features grouped by task type. Within each cognitive test, the measured variables exhibit strong positive correlations, often in the range of 0.8 to 0.95, indicating high internal consistency among metrics within the same task.

For example, the mean, variance, and attempt times in the Reaction Speed Tests and Decision-Making Tests correlate strongly with each other, suggesting that these features capture the same underlying cognitive speed component. This coherence is also observed in the Spatial Perception Test metrics, where time-based features cluster closely together.

Across different cognitive tests, the correlations are moderate (approximately 0.5–0.7), reflecting a shared latent factor related to general cognitive speed or executive function. For instance, reaction time variables from the Reaction Speed Tests, Decision-Making Test, and Verbal Memory and Working Memory Capacity Test exhibit moderate positive correlations, indicating that individuals who are slower in one task tend to be slower across other tasks as well. However, accuracy and correctness measures show weaker or even negative correlations across tasks, suggesting that they capture task-specific processes rather than a unified construct.

A key observation involves the relationship between task performance metrics and the Age variable. Age correlates positively with various timing measures and negatively with correctness scores. Typically, timing features such as total or mean attempt time correlate around 0.4 to 0.7 with age, while correctness metrics exhibit correlations between −0.3 and −0.6. This pattern indicates that older participants tend to perform tasks more slowly and slightly less accurately, which is consistent with known effects of aging on psychomotor and cognitive processing speed.

Another noteworthy aspect is the presence of very strong correlations between some feature pairs (greater than 0.9), signaling significant redundancy and potential multicollinearity. In the context of regression or predictive modeling, such highly interrelated variables would need to be addressed carefully; for example, through feature selection.

This correlation matrix highlights several important patterns: cognitive task measures show high internal coherence within each domain, moderate shared variance across tasks pointing to global cognitive processing effects, and clear age-related trends in both speed and accuracy. The visualization thus provides a comprehensive overview of how diverse cognitive and motor features relate to each other and how they jointly reflect broader aspects of cognitive aging.

**Figure A8:**
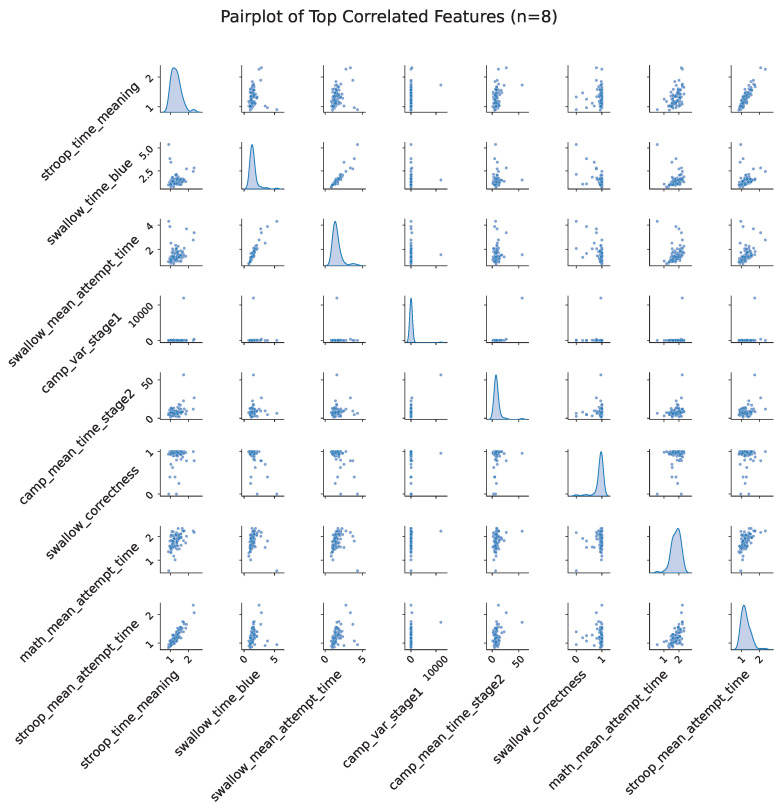
Matrix of plots showing the pairwise relationships between highly correlated variables in the dataset.

We plotted the most highly correlated pairs of features (correlation values greater than 0.5) in a matrix plot, showing the pairwise relationships between variables in the dataset (Figure A8). Among all the features, eight were identified as the most highly correlated:

1. stroop_time_meaning
2. swallow_time_blue
3. swallow_mean_attempt_time
4. camp_var_stage1
5. camp_mean_time_stage2
6. swallow_correctness
7. math_mean_attempt_time
8. stroop_mean_attempt_time

These variables reflect the results of several tests, including the Verbal Memory and Working Memory Capacity Tests, Decision-Making Tests, Color Campimetry Tests, and Spatial Perception Tests. The results of the Reaction Speed Test were the least correlated relative to the other features. The plot reveals strong positive correlations among time-based features and negative correlations between timing and correctness measures.

Additionally, a correlation analysis of the variables with age was conducted to reflect the strength of each variable’s association with age (Table A2 in the Appendix A). The highest positive correlation is observed for the variable stroop_time_color, with a value of approximately 0.645, indicating a strong direct relationship—completion time for this task increases with age.

Following this, the Stroop test time features, the mean and variance of trial times, and the time to complete math problems all show positive correlations ranging from approximately 0.3 to 0.5, indicating that these measures also increase with age. Mean completion times and several other memory and attention test parameters demonstrate positive correlations between 0.2 and 0.4, further suggesting a direct relationship with age.

Conversely, some indicators, such as answer accuracy in math and other tests, as well as gender (sex_male), show negative correlations with age, ranging from −0.06 to approximately −0.35. This implies that accuracy decreases with age, and the negative correlation with gender indicates that this variable changes inversely with age.

Several methods were subsequently applied to reduce multicollinearity among features and for feature selection (Table 3).

To determine the optimal feature selection method for this problem, multiple approaches were applied to the regression algorithms used in this study, and their performance was evaluated using mean absolute error (MAE), $R^{2}$, and $CV\text{\_}R^{2}$ metrics (see Table A3 in the Appendix A). Feature selection using SHAP, Permutation Importance, and SelectFromModel was implemented with a Random Forest model.

The results demonstrate that the SHAP and Permutation Importance methods yield superior performance, with $R^{2}$ values of approximately 0.63–0.66 and MAE values of approximately 6–7, indicating strong predictive capability. In particular:

- **SHAP** with 10 features achieves MAE ≈ 6.29 and $R^{2}\approx$ 0.62–0.63
- **Permutation Importance** with 10 features achieves MAE ≈ 6.14 and $R^{2}\approx$ 0.62–0.66

In contrast, the VIF and RFE methods show significantly poorer performance, with $R^{2}$ values ranging from approximately −0.46 to 0.5 and MAE values exceeding 11. The negative $R^{2}$ values obtained with VIF-based approaches indicate either insufficient predictive power with the selected features or persistent multicollinearity issues. For further analysis, the features selected using the Permutation Importance method were employed.

### 3.2. Model Training and Testing

To select the optimal model, several regression algorithms were employed: Random Forest Regressor, Extra Trees Regressor, Gradient Boosting Regressor, Support Vector Regression (SVR), Linear Regression, Lasso Cross-Validated (LassoCV), Ridge Cross-Validated (RidgeCV), Elastic Net Cross-Validated (ElasticNetCV), Adaptive Boosting Regressor (AdaBoost), and Bagging Regressor (Bagging).

The following algorithms and parameters were used for training and testing:

For linear models, LinearRegression was used with default parameters; LassoCV with alphas ([0.1, 1.0, 10]) and 5-fold cross-validation (cv = 5); RidgeCV with alphas ([0.1, 1.0, 10]) and cv = 5; and ElasticNetCV with l1_ratio ([0.1, 0.5, 0.9]) and cv = 5.

For ensemble methods, RandomForestRegressor, GradientBoostingRegressor, AdaBoostRegressor, ExtraTreesRegressor, and BaggingRegressor were trained using parameter search via GridSearchCV on the specified grids:

- **RandomForestRegressor**: n_estimators = [100, 200], max_depth = [None, 10, 20, 30], min_samples_split = [2, 5, 10], min_samples_leaf = [1, 2, 4], max_features = [“auto”, “sqrt”, “log2”].
- **GradientBoostingRegressor**: n_estimators = [100, 200, 300], learning_rate = [0.01, 0.05, 0.1], max_depth = [3, 5, 10], min_samples_split = [2, 5, 10].
- **AdaBoostRegressor**: n_estimators = [50, 100], learning_rate = [0.01, 0.05, 0.1] to reduce the risk of overfitting.
- **ExtraTreesRegressor**: n_estimators = [100, 200], max_depth = [5, 10, 20], min_samples_ split = [2, 5, 10], min_samples_leaf = [1, 2, 4], max_features = [“sqrt”, “log2”].
- **BaggingRegressor**: n_estimators = [10, 50, 100], max_samples = [0.5, 0.7, 1.0], max_features = [0.5, 0.7, 1.0] to limit the number of samples and features.
- **Support Vector Regression (SVR)**: C = [0.1, 1, 10] and kernel = [‘rbf’, ‘linear’] were used.

RepeatedKFold with 5 folds and 2 repetitions (random_state = 10) was used as the cross-validation strategy. Models with parameter grids were trained using GridSearchCV optimized for the ($R^{2}$) metric. Models without parameter grids were trained directly on the data.

The results were assessed using MAE and ($R^{2}$) metrics on the training and test sets, and the average cross-validated $R^{2}$ ($CV\text{\_}R^{2}$) was calculated. Additionally, bootstrapping was performed to estimate confidence intervals for the metrics.

**Figure 2:**
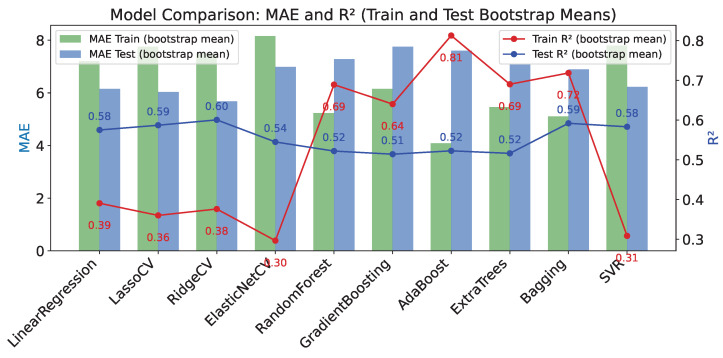
Comparison of Model Performance on Training and Test Sets Based on Bootstrap Mean MAE and $R^{2}$.

Based on a comparative analysis of the machine learning results for the regression problem, the following conclusions can be drawn about the performance of the various algorithms (Figure 2). Model evaluation results are presented using bootstrapped mean metric values and their 95% confidence intervals for both the training and test sets (Table A4 in Appendix A).

For the **LinearRegression** model, the mean error (MAE) during training is 7.203±[5.825,8.710], RMSE is 9.378±[7.553,11.378], and the coefficient of determination is $R^{2}=0.391\pm \left[0.173,0.563\right]$. On the test set, MAE and RMSE are lower: 6.156±[3.617,8.777] and 8.355±[5.190,11.558], respectively, with $R^{2}=0.575\pm \left[0.046,0.873\right]$, indicating better predictive ability on the test set, although the confidence interval for $R^{2}$ is quite wide.

The **LassoCV** model shows similar results: on training MAE=7.756±[6.437,9.158], $R^{2}=0.360\pm \left[0.202,0.489\right]$; on testing MAE=6.035±[3.605,8.933], $R^{2}=0.587\pm \left[0.161,0.880\right]$, indicating competitive performance with LinearRegression.

**RidgeCV** shows better test quality with MAE=5.681±[3.322,8.660], RMSE=7.894±[4.647,11.267], and $R^{2}=0.601\pm \left[0.164,0.889\right]$, demonstrating more stable and higher results.

**ElasticNetCV** has higher errors and lower quality: training MAE around 8.159, test MAE 6.984, and test $R^{2}=0.545\pm \left[0.203,0.777\right]$.

Ensemble models perform better during training. For example, **RandomForest** has high training accuracy: MAE=5.235±[4.222,6.323], $R^{2}=0.689\pm \left[0.616,0.759\right]$, but reduced test accuracy: MAE=7.283±[4.722,10.104], $R^{2}=0.522\pm \left[0.221,0.726\right]$, indicating overfitting.

**GradientBoosting** and **ExtraTrees** show similar patterns, with average test $R^{2}$ around 0.51–0.52 and relatively high test errors.

**AdaBoost** shows very high accuracy on training with $R^{2}=0.813\pm \left[0.719,0.888\right]$, but on testing $R^{2}$ decreases to around 0.523 with a wide confidence interval, which also indicates significant overfitting.

**Bagging** shows balanced results with $R_{\mathrm{train}}^{2}=0.719\pm \left[0.631,0.788\right]$ and $R_{\mathrm{test}}^{2}=0.592\pm \left[0.358,0.767\right]$, which is one of the best balances of quality and robustness.

**SVR** for $R^{2}$ results and errors is approximately at the level of linear models, with $R_{\mathrm{test}}^{2}=0.583\pm \left[0.253,0.838\right]$.

Average cross-validation $R^{2}$ (CV_R^2^) values range from negative for some linear models (e.g., LinearRegression around −0.205) to positive values for ensembles (up to 0.238 for RandomForest), further confirming the superior generalization ability of ensemble methods.

Thus, ensemble methods (RandomForest, Bagging, GradientBoosting) provide higher accuracy in training but are prone to overfitting, while regularized linear models (RidgeCV, LassoCV) show more balanced and stable test results with reasonable errors and good $R^{2}$ values.

### 3.3. Feature Importance Assessment in the RidgeCV Model

**Figure 3:**
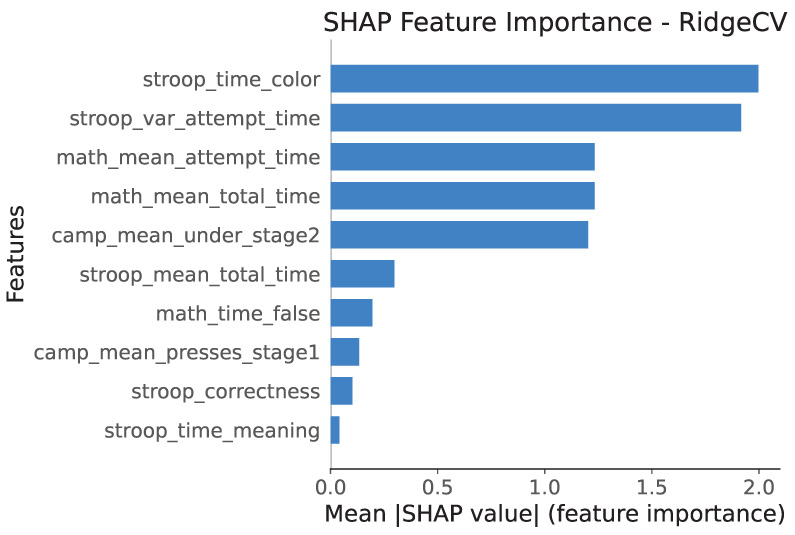
SHAP analysis for RidgeCV model.

Using SHAP (SHapley Additive exPlanations), feature importance was assessed through the mean absolute SHAP value for each feature, reflecting its average contribution to the deviation of model predictions from the baseline (Figure 3).

These results indicate that the stroop_time_color and stroop_var_attempt_time features have the greatest impact on the model’s predictions, with maximum mean absolute SHAP values of approximately 2.0, indicating strong contributions to the final prediction.

Features such as math_mean_attempt_time, math_mean_total_time, and camp_mean_ under_stage2 show mean SHAP values of approximately 1.2, representing significant but secondary importance compared to the top two features.

The low SHAP values of the remaining features indicate their relatively minor contributions to the model’s predictions.

The permutation importance method was also used to assess feature importance (Table 4). This method measures the impact of each feature on prediction quality by repeatedly shuffling its values and assessing the degradation in performance metrics. For each feature, the mean importance value and standard deviation are calculated across iterations, allowing us to evaluate feature significance for the model.

Formally, the permutation importance of feature *j* is the average change in the model’s performance metric when randomly shuffling the values of feature *j*, breaking its connection with the target variable, while all other features remain unchanged. In our case, importances are expressed as mean values with confidence intervals, that is,

(5) $${\mathrm{importance}}_{j}=\mu_{j}\pm \sigma_{j},$$

where $\mu_{j}$ is the average impact on the metric after permuting feature *j*, and $\sigma_{j}$ is the standard deviation of the score across different permutations.

This means that the stroop_time_color and stroop_var_attempt_time features have the greatest impact on the quality of the model: swapping them causes the most noticeable degradation in the metric. Features with low or negative importance have virtually no effect on the model or may even slightly improve performance when permuted, indicating that they are uninformative for the given task.

Thus, importances demonstrate which features are key to prediction and allow one to focus on the most informative features, while excluding or examining less important features separately to improve the quality and interpretability of the model.

**Figure 4:**
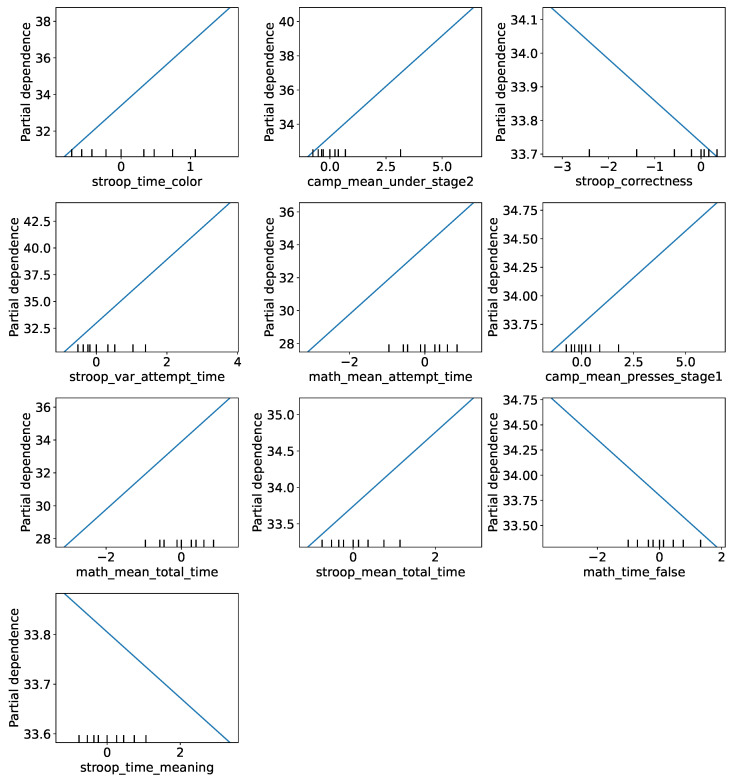
Partial Dependence Analysis for RidgeCV model features.

Partial Dependence results (Figure 4) demonstrate how the model’s mean prediction changes with varying values of a selected feature, averaged across all other features.

For the stroop_time_color feature, a monotonic increase in the model’s mean prediction is observed, from approximately 30.6 at values around −0.82 to approximately 38.75 at values around 1.57. This suggests that an increase in this feature’s value is associated with an increase in the predicted target.

Similarly, for the camp_mean_under_stage2 feature, the mean prediction increases from approximately 32.1 at values around −0.99 to approximately 40.8 at values around 6.40, demonstrating a strong positive impact on prediction.

The stroop_correctness feature has a less pronounced impact, with the average predicted value gradually decreasing from approximately 34.14 at values of −3.24 to approximately 33.69 at values of 0.34, suggesting some inverse correlation.

For stroop_var_attempt_time, the average predicted value increases from approximately 30.28 to 44.23 as the feature increases from approximately −0.91 to 3.78, indicating a significant impact on the prediction.

The model’s predicted values consistently increase for the features math_mean_attempt_ time, camp_mean_presses_stage1, math_mean_total_time, and stroop_mean_total_time, where changes in the values of these features are accompanied by an increase in the average prediction, but with a smaller slope compared to the first features.

For the math_time_false and stroop_time_meaning features, the relationship is less straightforward, and changes in the mean forecast are less pronounced.

**Figure 5:**
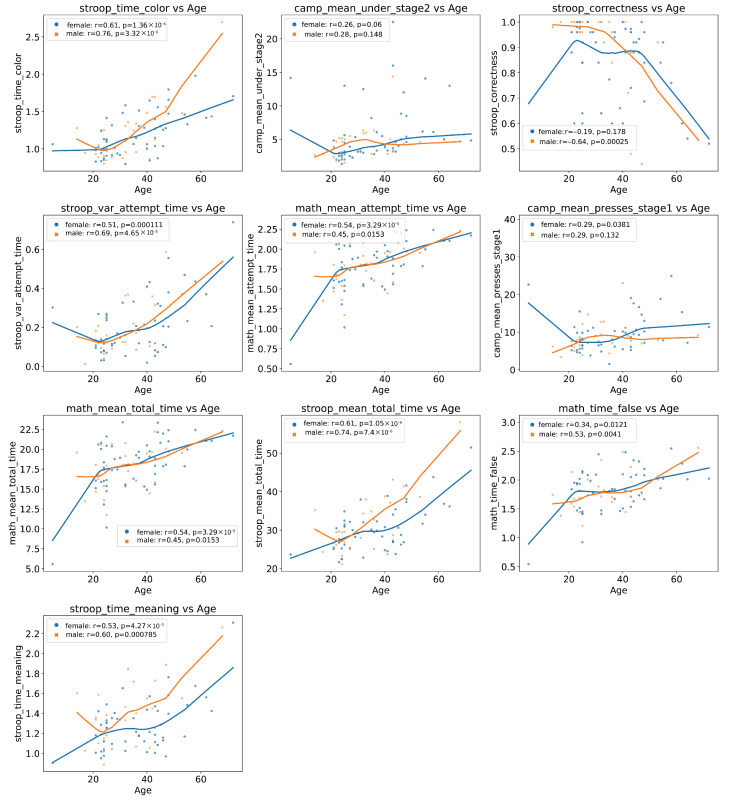
Scatter plots of each feature RidgeCV model against age with Pearson correlation coefficients with age (r) along and corresponding *p*-values for stratification by sex.

Next, scatter plots of each feature against age were constructed to assess linearity/non-linearity assumptions, and Pearson correlation coefficients with age along and corresponding *p*-values were calculated to test relationship significance (Figure 5).

The results of the correlation analysis of the features with age, adjusted for gender, show significant gender differences in the strength and direction of the associations.

The stroop_time_color feature has a strong positive correlation in both women (r=0.608, $p=1.36\times 10^{-6}$) and men (r=0.756, $p=3.32\times 10^{-6}$), with the association being stronger in men.

stroop_var_attempt_time also shows a significant association with age: in women (r=0.506, p=0.000111), and an even stronger one in men (r=0.691, $p=4.65\times 10^{-5}$).

stroop_mean_total_time showed a similar pattern: a strong positive correlation in women (r=0.613) and men (r=0.738).

The math_mean_attempt_time and math_mean_total_time features have a moderate positive correlation, slightly stronger in women (around 0.54) and less pronounced, but significant, in men (around 0.45).

math_time_false and stroop_time_meaning also correlate positively with age in both groups, with moderate *r* values and significant *p* values.

Interestingly, stroop_correctness shows a negative correlation with age, which is strong and statistically significant in men (r=−0.639, p=0.00025), but weaker and non-significant in women.

For the camp_mean_under_stage2 and camp_mean_presses_stage1 features, the correlation is weak and statistically insignificant for men, and weak but tending toward significance for women (*p* values around 0.04–0.06).

Thus, most features demonstrate a consistent relationship with age, with the relationship often stronger for men.

## 4. Discussion

Machine-learning approaches to age estimation from psychophysiological data have shown strong potential across cognitive, physiological, and neuroimaging modalities. In the present study, cognitive-age prediction models based on psychophysiological tests were optimized through rigorous preprocessing, outlier control, and explainability-driven feature selection. Winsorization effectively stabilized reaction-time variables and reduced extreme-value influence without shrinking the dataset, improving robustness of subsequent models. Model-based interpretability tools—SHAP and permutation importance—outperformed classical feature-screening methods by isolating the most relevant cognitive markers, particularly Stroop performance and working-memory features that align with established theories of age-related slowing in executive function.

Regularized linear models (RidgeCV, LassoCV) achieved the best generalization in this dataset, outperforming higher-variance ensemble approaches. RidgeCV combined with curated features achieved MAE ≈ 5.7 years with ($R^{2}\approx 0.60$), indicating that linear models can be efficient and stable for moderately sized psychophysiological datasets.

Across the broader literature, however, nonlinear and ensemble models often dominate age-estimation tasks in high-dimensional physiological data. XGBoost and Random Forests achieve state-of-the-art results in neuroimaging-based brain-age prediction, reaching MAEs of 1.49–1.58 years [46]. Support Vector Regression with nonlinear kernels also performs strongly, with MAEs of 1.64 years in youth MRI studies and 4.63 years ($R^{2}$ = 0.88) in adult brain-age work [46,47]. EEG-based age prediction similarly benefits from nonlinear models: ensemble and deep-learning approaches yield R^2^ values up to 0.37 in adults [48], MAE = 1.22 years in children using random forests [49], and MAE ≈ 1 month in infant deep-learning models [50]. Other physiological modalities show parallel trends- SVMs and GMMs perform well in spirometry (MAE = 6.35–6.54 [51]), CNNs excel in ECG-based age prediction (MAE = 1.49; $R^{2}$ = 0.53 [52]), and facial-image CNNs achieve accuracies near 98% ([53]). Although elastic-net regression is less flexible, it provides a favorable efficiency–accuracy balance across multiple signal types (MAE = 2.75–10.50 [54]).

Comparatively, the current study’s findings highlight that when sample sizes are limited and multicollinearity is high—as common in psychophysiological testing—regularized linear models combined with targeted feature selection can outperform more complex methods. Gender-stratified analyses supported demographic modulation of feature–age patterns, underscoring the importance of covariate-aware modeling. Limitations remain, including the cross-sectional design and restricted cohort size, mirroring broader field-wide challenges such as demographic confounds, variability in data acquisition, and interpretation of the predicted–chronological “age gap”. Prospective longitudinal studies are essential to validate predictive capacity over time and across diverse populations. Integration with neurobiological biomarkers and neuroimaging variables may also enrich model interpretability and clinical relevance.

Integrating robust preprocessing, explainability-guided feature selection, and linear regularization yields a practical and interpretable framework for cognitive-age estimation from psychophysiological tests. When situated within the broader literature—where XGBoost, Random Forests, SVR, and CNNs achieve leading performance across physiological signals—this work demonstrates that scalable cognitive-health screening can be achieved even without complex nonlinear models, provided the data are carefully curated and interpreted [46,47,48,49,50,51,52,53,54,55].

Of the 10 parameters selected, eight reflect the speed and quality of information processing, as well as attention span, while two indicators relate to the ability to distinguish colors and respond flexibly to changing stimuli (Table A5 in Appendix A). From a biological perspective, the speed and quality of information processing and sustained attention can be combined into the concept of the efficiency of cognitive processes, a leading indicator of which is cognitive control. A recent meta-analysis demonstrated a significant correlation between cognitive control and age [56]. Cognitive control is the cognitive processes that allow people to manage and regulate their attention, thoughts, and actions. It plays an important role in goal-directed behavior, allowing us to focus on a goal and quickly solve problems. Color perception processes also correlate strongly with chronological age [29]. Thus, our data are fully consistent with the extensive literature from recent reviews.

## 5. Conclusions

The present study identified the RidgeCV model with winsorization and standardization preprocessing as the best-performing algorithm for predicting cognitive age, achieving a mean absolute error of approximately 5.7 years and an $R^{2}$ of around 0.60 on test data. Feature selection guided by permutation importance and SHAP values highlighted key predictive features, notably Stroop test timings (stroop_time_color, stroop_var_attempt_time), and metrics from the math and color campimetry tests. These psychophysiological assessments, which measure executive function, processing speed, working memory, and visual perception, were most strongly associated with cognitive aging.

The findings emphasize the importance of rigorous outlier handling, multicollinearity-aware feature selection, and interpretable regularized regression models in analyzing psychophysiological data for cognitive aging. This approach enables sensitive and stable age predictions while identifying cognitive domains most affected by aging, thereby supporting scalable cognitive health screening and monitoring. Future work should pursue longitudinal validations and integration with neurobiological markers to enhance predictive power and clinical utility.

## Figures and Tables

**Table 1:** Psychophysiological tests with descriptions and variables.

Test Name	Description	Variables
Reaction Speed Test	Participants judge 10 arithmetic expressions as correct or incorrect by pressing green or red buttons.	Average time, time per attempt, variance, overall accuracy, accuracy on correct/incorrect inequalities.
Verbal Memory and Working Memory Capacity Test	Volunteers memorize 6 words, then identify words shown on screen as from the list or not.	Average time, time per attempt, variance, overall accuracy.
Decision-Making Test (Stroop Test)	Participants select colors based on letter color or word meaning to test cognitive switching and conflict resolution.	Average time, time per decision, variance, overall accuracy, correctness of meaning and color responses, decision times by section.
Spatial Perception Test	Respond to schematic swallow’s “flight” direction on colored backgrounds requiring same or opposite direction responses.	Average time, time per attempt, variance, overall accuracy, accuracy for “flying from” and “flying to” directions, decision times for each.
Verbal Function Test (Munsterberg Test)	Search for nouns hidden in random letter sequences within 1 min, recording number and proportion found.	Average time, time per word, variance, number of words found, proportion of words found.
Color Campimetry Test	Identify when animal shapes become visible or invisible against changing hues by pressing buttons.	Average times and variances for stages 1 and 2, including times for figure visibility and invisibility.

**Table 2:** Model performance for winsorization method with standardization (Z-score) (Winsor_standard) and robust preprocessing (Robust). Model parameters: RidgeCV (cv = 5), ElasticNetCV (cv = 5, random_state = 10, max_iter = 10,000, tol = 0.0001).

Model	MAE	R^2^	CV_R^2^
Winsor_standard			
RidgeCV	7.5460	0.5575	−0.5868
ElasticNetCV	8.5215	0.3977	−0.0536
Robust			
RidgeCV	7.6580	0.5169	−242.4276
ElasticNetCV	11.3410	−0.1227	−0.5374

**Table 3:** Feature selection methods with description.

Method	Description
VIF (Variance Inflation Factor)	Calculates the variance inflation factor and detects multicollinearity. Features with high VIF are removed [40,41].
SHAP (SHapley Additive exPlanations)	Evaluates the contribution of each feature to the prediction of a model based on cooperative game theory [42].
RFE (Recursive Feature Elimination) with Linear Regression	Iteratively removes the least significant features based on model weights [43].
SelectFromModel with Random Forest	Uses built-in random forest feature importance scores for threshold selection [44].
Permutation Importance	Evaluates the degradation of model quality when features are randomly shuffled [45].

**Table 4:** Permutation importance of feature with mean ± standard deviation.

Feature	Value
stroop_time_color	0.1250 ± 0.0292
stroop_var_attempt_time	0.1081 ± 0.0267
camp_mean_under_stage2	0.0631 ± 0.0195
math_mean_attempt_time	0.0522 ± 0.0240
math_mean_total_time	0.0522 ± 0.0240
stroop_mean_total_time	0.0052 ± 0.0037
camp_mean_presses_stage1	0.0027 ± 0.0020
stroop_correctness	0.0005 ± 0.0017
stroop_time_meaning	−0.0001 ± 0.0006
math_time_false	−0.0008 ± 0.0035

## Data Availability

The database used and analyzed during the current study is available from the corresponding author due to reasonable request because the database may contain personalized data related to subjects.

## Supplementary Materials

The following supporting information can be downloaded at: https://www.mdpi.com/article/10.3390/healthcare13243193/s1.

## Appendix A. Additional Graphs and Tables

**Table A1:** Variables Obtained from Psychophysiological Tests.

Variable Description	Variable Name	M ± Std (n = 99)	M ± Std (Male) (n = 67)	M ± Std (Female) (n = 32)	Units
**Swallow Test**					
Mean completion time	swallow_mean_total_time	49.72 ± 15.96	54.53 ± 12.61	50.88 ± 14.92	s
Mean time per attempt	swallow_mean_attempt_time	1.56 ± 0.69	1.45 ± 0.55	1.58 ± 0.62	s
Attempt time variability (variance)	swallow_var_attempt_time	0.95 ± 2	0.55 ± 1.53	0.92 ± 1.8	s
Attempt time deviation (std)	swallow_std_attempt_time	0.74 ± 0.63	0.58 ± 0.47	0.76 ± 0.59	s
Overall correctness	swallow_correctness	0.9 ± 0.2	0.94 ± 0.13	0.91 ± 0.2	
Red color correctness	swallow_correctness_red	0.88 ± 0.27	0.94 ± 0.18	0.89 ± 0.25	
Blue color correctness	swallow_correctness_blue	0.94 ± 0.16	0.97 ± 0.06	0.93 ± 0.18	
Mean attempt time (red)	swallow_time_red	1.59 ± 0.66	1.52 ± 0.71	1.61 ± 0.59	s
Time std (red)	swallow_time_red_std	0.73 ± 0.66	0.62 ± 0.64	0.78 ± 0.62	s
Mean attempt time (blue)	swallow_time_blue	1.52 ± 0.78	1.38 ± 0.41	1.53 ± 0.71	s
Time std (blue)	swallow_time_blue_std	0.66 ± 0.76	0.5 ± 0.25	0.66 ± 0.69	s
**Arithmetic Test**					
Mean completion time	math_mean_total_time	17.86 ± 3.1	17.84 ± 2.64	18.18 ± 3.25	s
Mean time per attempt	math_mean_attempt_time	1.79 ± 0.31	1.78 ± 0.26	1.82 ± 0.33	s
Attempt time variability (variance)	math_var_attempt_time	0.4 ± 0.25	0.4 ± 0.24	0.4 ± 0.27	s
Attempt time deviation (std)	math_std_attempt_time	0.6 ± 0.2	0.6 ± 0.19	0.6 ± 0.2	s
Overall correctness	math_correctness	0.82 ± 0.15	0.83 ± 0.16	0.81 ± 0.16	
Correctness (true inequalities)	math_correct_true	0.8 ± 0.19	0.79 ± 0.2	0.8 ± 0.2	
Correctness (false inequalities)	math_correct_false	0.85 ± 0.18	0.85 ± 0.19	0.82 ± 0.21	
Mean time (true)	math_time_true	1.74 ± 0.44	1.73 ± 0.33	1.79 ± 0.45	s
Time std (true)	math_time_true_std	0.55 ± 0.26	0.56 ± 0.33	0.57 ± 0.26	s
Mean time (false)	math_time_false	1.81 ± 0.36	1.82 ± 0.32	1.85 ± 0.38	s
Time std (false)	math_time_false_std	0.57 ± 0.25	0.6 ± 0.28	0.58 ± 0.24	s
**Munsterberg Test**					
Mean completion time	munster_mean_total_time	41.07 ± 10.57	41.05 ± 11.27	41.07 ± 10.75	s
Mean time per attempt	munster_mean_attempt_time	6.31 ± 2.85	6.6 ± 2.66	6.39 ± 2.77	s
Attempt time variability	munster_var_attempt_time	30.27 ± 39.64	29.17 ± 41.53	34.26 ± 40.03	s
Attempt time deviation (std)	munster_std_attempt_time	4.61 ± 3.03	4.48 ± 3.07	5.01 ± 3.05	s
Words found	munster_mean_words_found	6.95 ± 1.77	6.53 ± 1.47	6.82 ± 1.72	
Fraction of words found	munster_correctness	0.88 ± 0.15	0.87 ± 0.15	0.87 ± 0.16	
**Stroop Test**					
Mean completion time	stroop_mean_total_time	30.47 ± 7.05	32.05 ± 7.42	30.07 ± 5.85	s
Mean time per attempt	stroop_mean_attempt_time	1.22 ± 0.28	1.28 ± 0.3	1.2 ± 0.23	s
Attempt time variability	stroop_var_attempt_time	0.19 ± 0.15	0.21 ± 0.14	0.2 ± 0.14	s
Attempt time deviation (std)	stroop_std_attempt_time	0.41 ± 0.15	0.43 ± 0.15	0.42 ± 0.15	s
Overall correctness	stroop_correctness	0.87 ± 0.16	0.88 ± 0.17	0.86 ± 0.15	
Meaning correctness	stroop_correct_meaning	0.91 ± 0.12	0.91 ± 0.14	0.9 ± 0.15	
Color correctness	stroop_correct_color	0.79 ± 0.34	0.81 ± 0.32	0.76 ± 0.33	
Mean time (meaning)	stroop_time_meaning	1.32 ± 0.3	1.4 ± 0.3	1.27 ± 0.26	s
Time std (meaning)	stroop_time_meaning_std	0.4 ± 0.17	0.44 ± 0.16	0.39 ± 0.19	s
Mean time (color)	stroop_time_color	1.18 ± 0.35	1.22 ± 0.38	1.2 ± 0.27	s
Time std (color)	stroop_time_color_std	0.34 ± 0.22	0.34 ± 0.2	0.39 ± 0.23	s
**Memory Test**					
Mean completion time	memory_mean_total_time	10.69 ± 2.04	11.87 ± 3.05	10.61 ± 1.86	s
Mean time per attempt	memory_mean_attempt_time	1.07 ± 0.2	1.19 ± 0.3	1.06 ± 0.19	s
Attempt time variability	memory_var_attempt_time	0.17 ± 0.15	0.21 ± 0.18	0.15 ± 0.14	s
Attempt time deviation (std)	memory_std_attempt_time	0.37 ± 0.18	0.41 ± 0.19	0.34 ± 0.17	s
Overall correctness	memory_correctness	0.95 ± 0.08	0.93 ± 0.11	0.96 ± 0.07	
**Campimetry Test**					
Mean completion time (stage 1)	camp_total_time_stage1	52.6 ± 26.96	62.78 ± 57.61	74.11 ± 198.02	s
Mean completion time (stage 2)	camp_total_time_stage2	50.55 ± 26.91	59.8 ± 55.3	53.83 ± 49.22	s
Mean attempt time (stage 1)	camp_mean_time_stage1	8.77 ± 4.49	10.46 ± 9.6	12.35 ± 33	s
Mean attempt time (stage 2)	camp_mean_time_stage2	8.43 ± 4.48	9.97 ± 9.22	8.97 ± 8.2	s
Attempt time variability (stage 1)	camp_var_stage1	33.36 ± 55.13	404.06 ± 2112.84	5721.36 ± 46552.32	s
Attempt time deviation (stage 1)	camp_std_stage1	4.73 ± 3.35	7.96 ± 18.75	13.7 ± 74.95	s
Attempt time variability (stage 2)	camp_var_stage2	29.56 ± 92.56	807.82 ± 4353.43	64.66 ± 363.45	s
Attempt time deviation (stage 2)	camp_std_stage2	4.13 ± 3.57	9.24 ± 27.31	4.56 ± 6.67	s
Mean presses (stage 1)	camp_mean_presses_stage1	9.05 ± 4.84	9.57 ± 6.62	9.3 ± 4.71	
Mean underpress (stage 2)	camp_mean_over_stage2	−3.08 ± 4.5	−5.93 ± 8.46	−2.06 ± 1.33	
Mean overpress (stage 2)	camp_mean_under_stage2	5.5 ± 4.04	5.12 ± 3.64	5.68 ± 4.24	
Hit rate (stage 2)	camp_hit_stage2	0.03 ± 0.07	0.03 ± 0.07	0.03 ± 0.06	
**Age**					
Target variable	age	34.46 ± 13.43			years
**Sex**					
male = 67 (68%), female = 32 (32%)	sex				

**Table A2:** Correlation of Features with Age. Features most strongly correlated with age are highlighted in green.

Feature	Correlation
stroop_time_color	0.645393
stroop_mean_total_time	0.626247
stroop_mean_attempt_time	0.626247
stroop_var_attempt_time	0.561404
math_mean_attempt_time	0.519443
math_mean_total_time	0.519443
stroop_time_meaning	0.505494
swallow_time_red	0.466490
memory_mean_total_time	0.449405
memory_mean_attempt_time	0.449405
math_time_false	0.398898
math_time_true	0.363121
munster_mean_attempt_time	0.331035
munster_var_attempt_time	0.281586
camp_mean_presses_stage1	0.276171
camp_mean_under_stage2	0.272294
math_var_attempt_time	0.239193
memory_var_attempt_time	0.209551
camp_total_time_stage1	0.190043
camp_mean_time_stage1	0.190043
camp_mean_time_stage2	0.150702
camp_total_time_stage2	0.150702
swallow_correctness_blue	0.146714
swallow_time_blue	0.144619
swallow_correctness	0.140194
swallow_mean_total_time	0.136553
swallow_correctness_red	0.121541
camp_var_stage1	0.078214
camp_var_stage2	0.074556
swallow_var_attempt_time	0.071613
math_correct_true	−0.061698
camp_hit_stage2	−0.094003
sex_male	−0.150476
math_correctness	−0.181952
munster_mean_words_found	−0.217208
math_correct_false	−0.218936
memory_correctness	−0.242283
stroop_correct_color	−0.253286
munster_correctness	−0.284141
stroop_correct_meaning	−0.331289
stroop_correctness	−0.354784

**Table A3:** Model Performance for Different Feature Selection Methods. MAE = mean absolute error, RF = Random Forest. Best method and model are highlighted in green.

Feature Selection Method	Model	MAE	R^2^	CV_R^2^	Num Features
VIF	LinearRegression	11.487132	−0.011207	−0.271605	9
VIF	LassoCV	11.340993	−0.122677	−0.192899	9
VIF	RidgeCV	11.060649	0.033739	−0.163932	9
VIF	ElasticNetCV	11.290789	−0.056564	−0.117723	9
VIF	RandomForest	11.906471	−0.140773	−0.090463	9
VIF	GradientBoosting	11.239240	−0.000890	−0.435411	9
VIF	AdaBoost	10.778374	0.068670	−0.045725	9
VIF	ExtraTrees	12.572353	−0.369192	−0.103983	9
VIF	Bagging	13.705882	−0.527049	−0.079172	9
VIF	SVR	11.188732	−0.131680	0.003830	9
SHAP	LinearRegression	7.146253	0.550681	0.085518	10
SHAP	LassoCV	6.287083	0.624531	0.250726	10
SHAP	RidgeCV	6.570456	0.600076	0.170375	10
SHAP	ElasticNetCV	6.726426	0.612186	0.233749	10
SHAP	RandomForest	7.742353	0.582841	0.223484	10
SHAP	GradientBoosting	7.153854	0.602167	0.057243	10
SHAP	AdaBoost	7.656903	0.590895	0.244850	10
SHAP	ExtraTrees	7.418824	0.595322	0.190480	10
SHAP	Bagging	8.611765	0.463011	0.152066	10
SHAP	SVR	10.517277	−0.036322	0.207175	10
RFE LinearRegression	LinearRegression	12.414642	−0.464931	−0.308877	10
RFE LinearRegression	LassoCV	11.184127	−0.127148	0.017625	10
RFE LinearRegression	RidgeCV	10.507438	0.000344	0.091916	10
RFE LinearRegression	ElasticNetCV	11.340993	−0.122677	0.079094	10
RFE LinearRegression	RandomForest	8.691176	0.321287	0.102812	10
RFE LinearRegression	GradientBoosting	7.293369	0.503892	0.089338	10
RFE LinearRegression	AdaBoost	9.483558	0.266718	0.072122	10
RFE LinearRegression	ExtraTrees	9.244706	0.191799	0.122879	10
RFE LinearRegression	Bagging	10.835294	0.129466	0.046387	10
RFE LinearRegression	SVR	10.471777	−0.138648	0.041319	10
SelectFromModel RF	LinearRegression	10.948097	−0.030999	−0.249418	22
SelectFromModel RF	LassoCV	7.464744	0.542976	0.207067	22
SelectFromModel RF	RidgeCV	7.799463	0.430147	−0.106611	22
SelectFromModel RF	ElasticNetCV	7.925152	0.486154	0.134158	22
SelectFromModel RF	RandomForest	8.458235	0.457824	0.219908	22
SelectFromModel RF	GradientBoosting	7.970273	0.497819	0.141981	22
SelectFromModel RF	AdaBoost	8.097739	0.445380	0.269909	22
SelectFromModel RF	ExtraTrees	7.940588	0.531125	0.147997	22
SelectFromModel RF	Bagging	8.135294	0.493537	0.141889	22
SelectFromModel RF	SVR	11.080334	−0.209465	0.059060	22
Permutation Importance RF	LinearRegression	6.143887	0.624303	0.166694	10
Permutation Importance RF	LassoCV	6.166796	0.629573	0.252601	10
Permutation Importance RF	RidgeCV	5.711072	0.663672	0.182320	10
Permutation Importance RF	ElasticNetCV	6.387706	0.629427	0.232177	10
Permutation Importance RF	RandomForest	7.582941	0.599978	0.273077	10
Permutation Importance RF	GradientBoosting	7.717461	0.621514	0.209122	10
Permutation Importance RF	AdaBoost	8.537162	0.510334	0.351497	10
Permutation Importance RF	ExtraTrees	7.270000	0.622351	0.223448	10
Permutation Importance RF	Bagging	7.100000	0.616541	0.225358	10
Permutation Importance RF	SVR	10.538338	−0.026361	0.201514	10

**Table A4:** Model Evaluation Results with Bootstrapped Metrics (95% CI).

Model	MAE	RMSE	R^2^	Split	CV R^2^
RidgeCV	7.504 ± [6.134, 8.952] 5.681 ± [3.322, 8.660]	9.546 ± [7.688, 11.516] 7.894 ± [4.647, 11.267]	0.376 ± [0.192, 0.523] 0.601 ± [0.164, 0.889]	Train Test	0.0097
Bagging	5.110 ± [4.224, 6.090] 6.893 ± [4.808, 9.319]	6.375 ± [5.224, 7.687] 8.372 ± [5.663, 11.099]	0.719 ± [0.631, 0.788] 0.592 ± [0.358, 0.767]	Train Test	0.2007
LassoCV	7.756 ± [6.437, 9.158] 6.035 ± [3.605, 8.933]	9.646 ± [7.935, 11.516] 8.184 ± [4.784, 12.054]	0.360 ± [0.202, 0.489] 0.587 ± [0.161, 0.880]	Train Test	−0.0730
SVR	7.800 ± [6.346, 9.484] 6.227 ± [3.732, 9.239]	10.067 ± [8.200, 12.096] 8.269 ± [5.000, 11.722]	0.309 ± [0.154, 0.446] 0.583 ± [0.253, 0.838]	Train Test	0.1492
LinearRegression	7.203 ± [5.825, 8.710] 6.156 ± [3.617, 8.777]	9.378 ± [7.553, 11.378] 8.355 ± [5.190, 11.558]	0.391 ± [0.173, 0.563] 0.575 ± [0.046, 0.873]	Train Test	−0.2049
ElasticNetCV	8.159 ± [6.783, 9.674] 6.984 ± [4.407, 9.783]	10.122 ± [8.232, 12.096] 8.798 ± [5.515, 12.278]	0.297 ± [0.156, 0.412] 0.545 ± [0.203, 0.777]	Train Test	−0.0542
AdaBoost	4.086 ± [3.372, 4.814] 7.602 ± [5.432, 9.720]	5.112 ± [4.417, 5.817] 8.676 ± [6.670, 10.618]	0.813 ± [0.719, 0.888] 0.523 ± [−0.086, 0.798]	Train Test	0.1968
RandomForest	5.235 ± [4.222, 6.323] 7.283 ± [4.722, 10.104]	6.679 ± [5.234, 8.267] 9.142 ± [5.732, 12.386]	0.689 ± [0.616, 0.759] 0.522 ± [0.221, 0.726]	Train Test	0.2378
ExtraTrees	5.459 ± [4.494, 6.374] 7.084 ± [4.469, 10.036]	6.733 ± [5.530, 8.156] 9.152 ± [5.704, 12.225]	0.690 ± [0.607, 0.753] 0.516 ± [0.233, 0.701]	Train Test	0.2362
GradientBoosting	6.155 ± [5.273, 7.151] 7.754 ± [5.478, 10.058]	7.219 ± [6.176, 8.244] 9.035 ± [6.992, 10.971]	0.640 ± [0.557, 0.708] 0.514 ± [0.192, 0.691]	Train Test	0.1960

**Table A5:** Description of Selected Features for Cognitive Age Prediction.

Feature	Description
1. Mean time per attempt (Color, Stroop)	The average decision time in the Stroop test variant where the participant must select the color of the letters, even when the word itself names a different color. Reflects information-processing speed and attentional focus.
2. Variability of time per attempt (Stroop)	The dispersion of time spent on each attempt in the Stroop test. Indicates the stability of cognitive control and the ability to sustain attention.
3. Mean time per attempt (Math)	Average time required to solve one inequality in the mathematical test. Reflects processing speed and attentional concentration.
4. Mean total test time (Math)	Total time required to complete the mathematical test. Indicates overall processing speed and attention.
5. Mean number of presses (Campimetry)	In the second phase of color campimetry, participants press a button until a figure becomes indistinguishable from the background. Continued pressing after disappearance reflects delayed inhibition and reduced responsiveness to changing stimuli.
6. Mean total test time (Stroop)	Total time required to complete the Stroop test. Reflects information-processing speed and attentional control.
7. Mean time per incorrect-inequality evaluation (Math)	Average time needed to decide that an inequality is incorrect. Indicates processing speed and attentional control.
8. Mean number of presses in phase 1 (Campimetry)	In the first phase of color campimetry, participants press until they can detect a figure slightly different in color from the background. Measures the ability to discriminate color shades.
9. Overall accuracy (Stroop)	Percentage of correct responses out of all attempts in the Stroop test. Indicates quality of information processing.
10. Mean time per attempt (Meaning, Stroop)	Average decision time in the Stroop test variant where the participant must select the meaning of the word rather than the color of the letters. Reflects processing speed and attentional focus.
