# Supplementary material for: Using Machine Learning Methods to Predict Cognitive Age from Psychophysiological Tests
Source: Healthcare (Basel). 2025 Dec 5;13(24):3193. doi: 10.3390/healthcare13243193 (PMC12733081; doi:10.3390/healthcare13243193)
Supplement: Supplementary file 1 [file healthcare-13-03193-s001.zip › healthcare-3974064-supplementary.pdf]

Информированное согласие

Ваше участие в исследовании предполагает: прохождение тестов на определение когнитивных способностей.

В ходе исследования ожидается получить результаты, которые могут быть применимы для улучшения когнитивных способностей людей, для повышения работоспособности и стрессоустойчивости.

Полученная в ходе исследования информация не будет содержать персональных данных участников. Эта информация будет использована в научно-исследовательских целях только обезличено. Ваши персональные данные не будут упомянуты где-либо в связи с результатами исследования. Все данные, собранные в ходе исследования, будут представляться только в общем массиве и будут доступны только исследовательской группе.

Соглашаясь на участие в исследовании Вы: даете согласие исследователям на ознакомление с Вашими персональными данными и их обработку в соответствии с действующим законодательством, а также на передачу такой информации третьим лицам, в случаях, предусмотренных законодательством РФ либо обезличено.

Нажимая на кнопку «Согласен» Вы даете согласие на обработку своих персональных данных, то есть совершение, в том числе, следующих действий: обработку (включая сбор, систематизацию, накопление, хранение, уточнение (обновление, изменение), использование, обезличивание, блокирование, уничтожение персональных данных), при этом общее описание вышеуказанных способов обработки данных приведено в Федеральном законе от 27.07.2006 № 152-ФЗ, а также на передачу такой информации третьим лицам, в случаях, установленных нормативными документами вышестоящих органов и законодательством.

Настоящее согласие действует бессрочно.

Настоящее согласие может быть отозвано Субъектом в любой момент письменным заявлением субъекта персональных данных.

Informed Consent

Your participation in this study includes: completing cognitive assessment tests.

The study is expected to yield results that may be applicable to improving cognitive abilities, performance, and stress resilience.

The information obtained during the study will not contain any personal data of participants. This information will be used for scientific research purposes only in an anonymized form. Your personal data will not be mentioned in connection with the study results. All data collected during the study will be presented in a general form and will be accessible only to the research team.

By agreeing to participate in the study, you: consent to the researchers accessing your personal data and processing it in accordance with applicable law, as well as to the transfer of such information to third parties, in cases stipulated by Russian law, or in an anonymized form.

By clicking the "I Agree" button, you consent to the processing of your personal data, including the following actions: processing (including collection, systematization, accumulation, storage, clarification (updating, modification), use, depersonalization, blocking, and destruction of personal data). A general description of the above data processing methods is provided in Federal Law No. 152-FZ of July 27, 2006. You also consent to the transfer of such information to third parties, in cases established by regulatory documents of higher authorities and legislation.

This consent is valid indefinitely.

This consent may be revoked by the Subject at any time by a written statement from the subject of the personal data.
